# Image Correlation Method for DNA Sequence Alignment

**DOI:** 10.1371/journal.pone.0039221

**Published:** 2012-06-27

**Authors:** Millaray Curilem Saldías, Felipe Villarroel Sassarini, Carlos Muñoz Poblete, Asticio Vargas Vásquez, Iván Maureira Butler

**Affiliations:** 1 Depto. de Ingeniería Eléctrica, Universidad de La Frontera, Temuco, Chile and Agriaquaculture Nutritional Genomic Center (CGNA), Comisión Nacional de Investigación Científica y Tecnológica- Gobierno Regional, La Araucanía, Genomics and Bioinformatics Unit. Temuco, Chile; 2 Depto. de Ingeniería Eléctrica. Universidad de La Frontera. Temuco. Chile; 3 Depto. de Ciencias Físicas. Universidad de La Frontera. Center for Optics and Photonics. Temuco. Chile; 4 Agriaquaculture Nutritional Genomic Center (CGNA), Comisión Nacional de Investigación Científica y Tecnológica- Gobierno Regional, La Araucanía, Genomics and Bioinformatics Unit. Temuco, Chile; University of Zurich, Switzerland

## Abstract

The complexity of searches and the volume of genomic data make sequence alignment one of bioinformatics most active research areas. New alignment approaches have incorporated digital signal processing techniques. Among these, correlation methods are highly sensitive. This paper proposes a novel sequence alignment method based on 2-dimensional images, where each nucleic acid base is represented as a fixed gray intensity pixel. Query and known database sequences are coded to their pixel representation and sequence alignment is handled as object recognition in a scene problem. Query and database become object and scene, respectively. An image correlation process is carried out in order to search for the best match between them. Given that this procedure can be implemented in an optical correlator, the correlation could eventually be accomplished at light speed. This paper shows an initial research stage where results were “digitally” obtained by simulating an optical correlation of DNA sequences represented as images. A total of 303 queries (variable lengths from 50 to 4500 base pairs) and 100 scenes represented by 100 x 100 images each (in total, one million base pair database) were considered for the image correlation analysis. The results showed that correlations reached very high sensitivity (99.01%), specificity (98.99%) and outperformed BLAST when mutation numbers increased. However, digital correlation processes were hundred times slower than BLAST. We are currently starting an initiative to evaluate the correlation speed process of a real experimental optical correlator. By doing this, we expect to fully exploit optical correlation light properties. As the optical correlator works jointly with the computer, digital algorithms should also be optimized. The results presented in this paper are encouraging and support the study of image correlation methods on sequence alignment.

## Introduction

Genomics is a discipline focused on studying living organism’s genetic material, looking primarily for functional and evolutionary relationships among them [1]. Sequence alignment compares two or more DNA, RNA or protein sequences and seeks the greatest amount of overlap or matching among them. Given that matching areas usually involve coding and regulatory regions and other functional features, the use of similarity allows us to infer functional or evolutionary relationships between sequences [1], [2]. Thus, alignment techniques must distinguish between matches due to biological similarities from chance similarities.

To solve this issue, Needleman & Wunsch [3] and Smith & Waterman [4] proposed the use of dynamic programming algorithms which have become the most implemented algorithms in a number of popular alignment programs, such as BLAST and others [5]. Additionally, several alignment methods have been suggested with a variety of differences among them, such as sequence size handling, level of achieved similarity, gap and mutation (mismatches) treatment, search type (global or local), speed, and required accuracy [1]. Probably, the most important issue in sequence alignment is the trade-off between accuracy and efficiency [6]. In general, highly accurate methods tend to be slow when analyzing big databases and fast algorithms often sacrifice sensitivity and confidence in match quality [7].Thus, developing highly effective and efficient alignment methods remains a challenge.

Didelot et al. [8] used a Monte Carlo Markov Chain method for inferring alignment recombination rates and localizing recombination events. However, this algorithm resulted impractical when searching large databases. To decrease computational burden, Rozanov et al. [9] provided a genotyping tool for viral genomes. Their algorithm was faster than Didelot et al.’s method, but less accurate. Other approaches, such as *k*-word composition based alignment-free methods [10] and heuristic techniques, such as genetic algorithms [11], tree data structure [12] and self-organizing neural network [13] have also been proposed. Although most sequence comparison methods are based on string matching, other comparison types are also possible. Visualization and graphical methods have been used to examine global similarities among coding sequences [12], [14]. 2-D and 3-D DNA graphical representations have allowed visual characterizations with low degeneracy [15], [16]. Randic et al. [15] proposed a method where graphs were obtained by assigning positive and negative x and y values (axes) to the four nucleotide bases. 2D visualization comparisons have also been accomplished by using dual base curves (DB-curve) [17], where two bases are assigned with +45° and −45° vectors, and the remaining bases with a +90° vector. Sequence similarities and differences can be readily observed; however, these representations show limitations when analyzing long sequences. Signal processing and pattern recognition methods require coding DNA sequences into numeric sequences. Anatassiou [18] showed that protein or DNA sequences can be mapped into one or more numerical sequences to obtain a clear signal. Genomic signal processing was defined by Dougherty et al. [19] and allows a variety of methods, such as the Fourier (FFT) [20], [21] and wavelet transformations [22] for solving relevant bioinformatics and computational biology problems.

The FFT incorporation using correlation methods for DNA sequence aligning, allowed obtaining very good results. Rockwood et al [7] proposed a cross-correlation method to compare sequences, applying the FFT to improve computational efficiency. Bases were represented as complex numbers. Their work demonstrated that the cross-correlation alignment method was able to detect even deeply hidden sequence homology that many existing alignment methods are unable to detect. Brodzik [23] compared the standard magnitude-and-phase cross-correlation technique with the phase-only cross-correlation technique and demonstrated that they are a robust way to isolate insertion/deletions and to identify matching segment positions. In this work DNA bases were represented as binary numbers and the sequence by a digital signal.

Our work’s aim is to evaluate the correlation process when four DNA nucleotides are transformed into four different numbers, respectively represented by four gray pixel intensities and visualized in a 2D image. Both sequences, the query and the known database are represented as images and compared using a computer simulated optical correlation technique [24], [25]. The main advantage of the proposal is if sequences are well recognized by the digital correlation process, the optical correlations occur at light speed. The idea was inspired by the fact that sequence comparison is often carried out visually by geneticists, taxonomists, and molecular biologists when small number of sequences are under study. The paper includes a set of simulations to evaluate the effectiveness and the limitations of this method. Many queries were carried out and the results compared to BLAST outputs.

## Materials and Methods

### Optical Correlation

Optical correlators are pattern recognition tools used to determine if an object is in an input scene. The similarity degree between input scene and object is evaluated by correlation peaks intensity. The peak location also gives information concerning object position in the scene. There are two main correlating architectures: the Vander- Lugt Correlator [26] and the Joint Transform Correlator [25]. The first requires a priori digital Fourier transformation of the object, or “filter”, whereas in the second architecture transformed images are obtained by an optical process. In this paper we will refer to the Vander- Lugt Correlator as the simulated optical correlator.

The correlation function between two signals *s(x, y)* and *o(x, y)*, is defined by the expression shown in Equation 1:
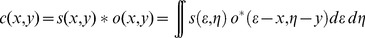
(1)Where {*} is the complex conjugated. The correlation function between two functions is maximum when these functions are identical, if one of these functions is the input scene *s(x,y)* and the other function corresponds to the object *o(x,y)*, one will have a maximum value when the object is present in the scene. If *S(u, v)* and *O(u, v)* are the Fourier Transformation of *s(x, y)* and *o(x, y)*, respectively, the correlation can be carried out in the frequency domain as stated by Equation 2:



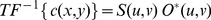
(2)The input scene, *S(u, v)* and the reference object, *O(u, v)* are described in the frequency domain by the Fourier Transformation [27]. The function O*(u, v), also called Classical Matched Filter (CMF) or mask, selects object information in the frequency domain by means of a filtering process.

The correlation process can be carried out in an optical correlator: the Fourier transformed input scene *S(u, v)* is obtained optically in a certain plane (image plane of light source plane) that holds the spectral information of the image (frequency domain). If the spectral information of the filter O*(u, v) is put in this plane, the correlation process occurs according to the right side of the equation 2. A second optical Fourier transform is necessary to obtain the correlation process in the spatial domain. This process can be computer simulated; however, the great advantage of an optical correlator is its capacity to make the Fourier transformation process and correlation at light speed. Once the optical system is aligned, transformation and correlation are immediate; thus, these may be useful in applications where time is critical or the amount of data is large.

**Figure 1:**
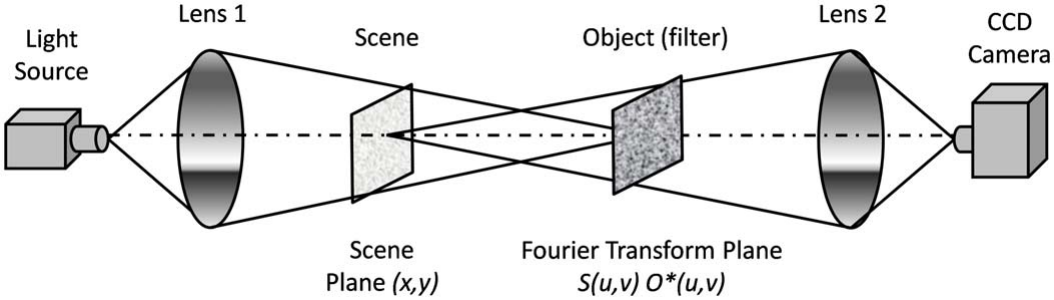
Convergent Vander- Lugt optical correlator. O is a monochromatic source light, L1 and L2 are lenses, the scene and the filter planes hold both images to correlate and a CCD camera registers the correlation plane. The Fourier transform of the input image *s(x,y)* is obtained at the Filter plane where the optical correlation S(u,v)H*(u,v) takes place. The correlation result *c(x,y)* is captured by the CCD camera.

To increase the processing speed, liquid crystal display (LCD) spatial light modulators are currently used in optical setups. In this case, the scene and the filter are displayed onto a LCD, modulating in amplitude and phase, respectively [28]. Figure 1 shows an optical correlation process schema. The correlation plane is formed at the correlators end, specifically, at the image scene-object plane of the second lens, where it is captured by a CCD camera.

This architecture requires that the object information be provided, as a filter, onto the Fourier Transform plane. The filter allows the modification of the information contained in the object’s image spectrum [29], [30]. Because of its good efficiency and simplicity, the Phase Only Filter (POF) has been widely studied in optical correlation [31], [32]. This filter optimizes the luminous efficiency criteria, which is extremely important in the image optical correlation processing, since it maximizes the amount of luminous energy at the correlation plane. The POF is defined in Equation 3.
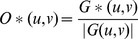
(3)Where *G(u, v)* is the Fourier Transformation of the object. Filters depend on this application, due to the fact that they optimize certain image characteristics [28].

### Sequences

The database was configured extracting one million NCBI DNA base pairs, corresponding to a section of the sequences 4,809,037 base pairs (bp): NC_003198.1 (*Salmonella enterica* subp. *enterica* serovar Typhi str. CT18 chromosome, complete genome). The database was divided into one hundred scenes with a size of 100 x 100, thus containing 10,000 bp each.

**Figure 2:**
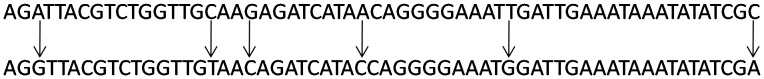
Creation of mutated sequences. Example of the 10% of mutation (6 bases) of a 60 bases sequence, creating a new sequence (bottom) from the original sequence (upper). The loci and the replacing bases are randomly chosen.

As in many studies [15] [22]–[23], queries were artificially generated in order to evaluate the effect of their correlation process variability (like their length, mismatched bases number and presence or absence in the database). Queries were implemented by extracting randomly 303 varying size sequences from 50 to 4,500 bp. The queries were represented as 303 objects. To simulate differences between the queries and the original sequences, new sequences were created by randomly substituting a percentage of bases. Base positions and new bases (substitutes) were randomly chosen. Figure 2 illustrates a 60 pb sequence mutation. The mutation process generated six new sets of 303 queries each, with 10 to 60% mutations (step 10), similar to noise addition in pattern recognition. Finally 2,121 query sequences were searched in a 1,000,000 bp database.

### Evaluation Criteria

Given that different alignment methodologies use different evaluation criteria, and that alignments were treated as a pattern recognition problem, we evaluated our methodology using standard pattern recognition criteria, the time needed, or efficiency, to compute the alignment.

Four statistical indexes, commonly used in classification processes, were used to measure recognition. Sensitivity (Se) measures the ability to detect true positives, in other words, to correctly identify an object when it is in the scene. Specificity (Sp) measures the ability to detect true negatives, in other words, to correctly detect that the object is not in the scene. Exactitude (Ex) computes all the correctly classified objects whereas Error (Er) computes the incorrectly classified objects. These indexes are calculated according to the following relationships and are expressed in percentages:
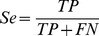
(4)

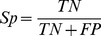
(5)

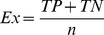
(6)

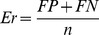
(7)where n is the total number of recognized elements; TP (true positives) is the number of objects correctly identified in a scene; TN (true negatives) is the number of objects correctly identified as not being in the scene; FP (false positives) and FN (false negatives) are object numbers wrongly identified as being or not being in a scene, respectively. The statistical indexes and the time were computed after the simulations were performed.

### Digital Image Correlation

**Figure 3:**
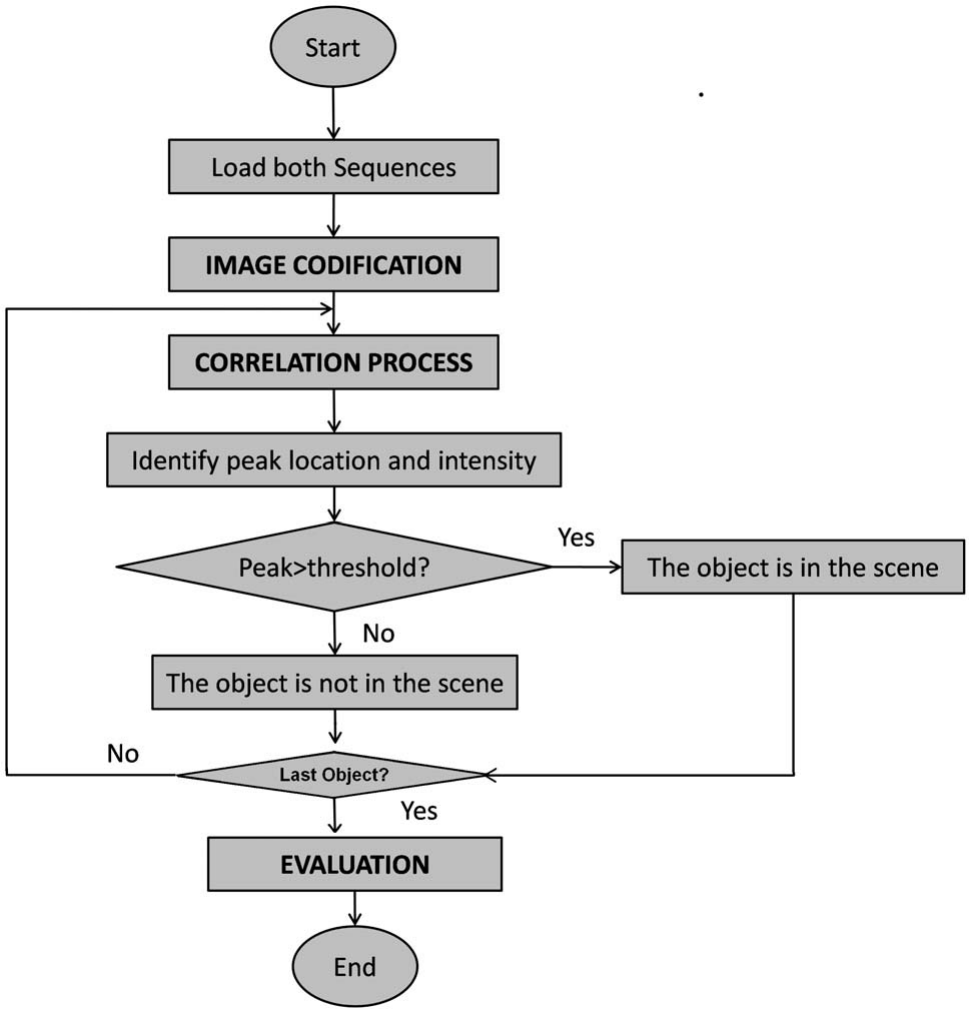
Algorithm of the digital correlator. The different steps of the digital correlation process are represented in the algorithm. This algorithm is executed for each pair object-scene. The output results are stored and the whole process is finally evaluated.

The whole correlation process was simulated in Matlab 7.1, using a 1.8-GHz Mobile AMD Semprom Portable Computer with 1.472 GB of memory, running on Microsoft Windows XP Professional Service Pack 3.The 2.2.24+.BLAST version was obtained from the NCBI website on October 2010. The algorithm presented in Figure 3 performed each object-scene recognition process. The analysis of the more time expensive algorithm steps shown in Figure 3 were performed using a MATLAB profiler.

#### a) Codification

**Figure 4:**
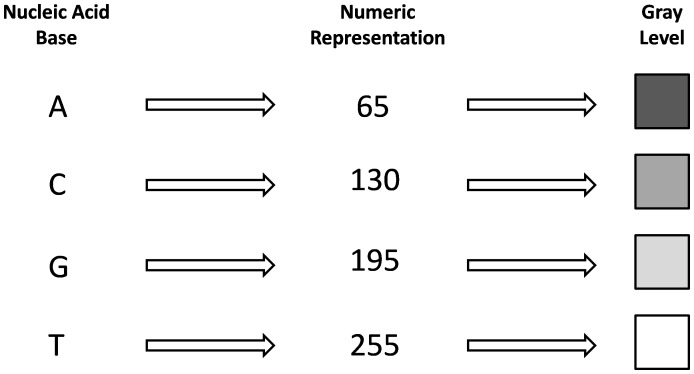
Base Codification. Each nucleic acid base is represented by a specific number which represents the gray level of a pixel.

Sequences were represented as images formed by four gray level pixels representing bases. Each nucleotide was coded by a numeric value ranging from 0 and 255, as shown in Figure 4. Each value represents a gray level in the grayscale, where zero corresponds to black and 255 corresponds to white. The values of Figure 4 were chosen because they represent a constant separation of the gray levels; however, future biological analysis may give more consistent numeric correspondences.

**Figure 5:**
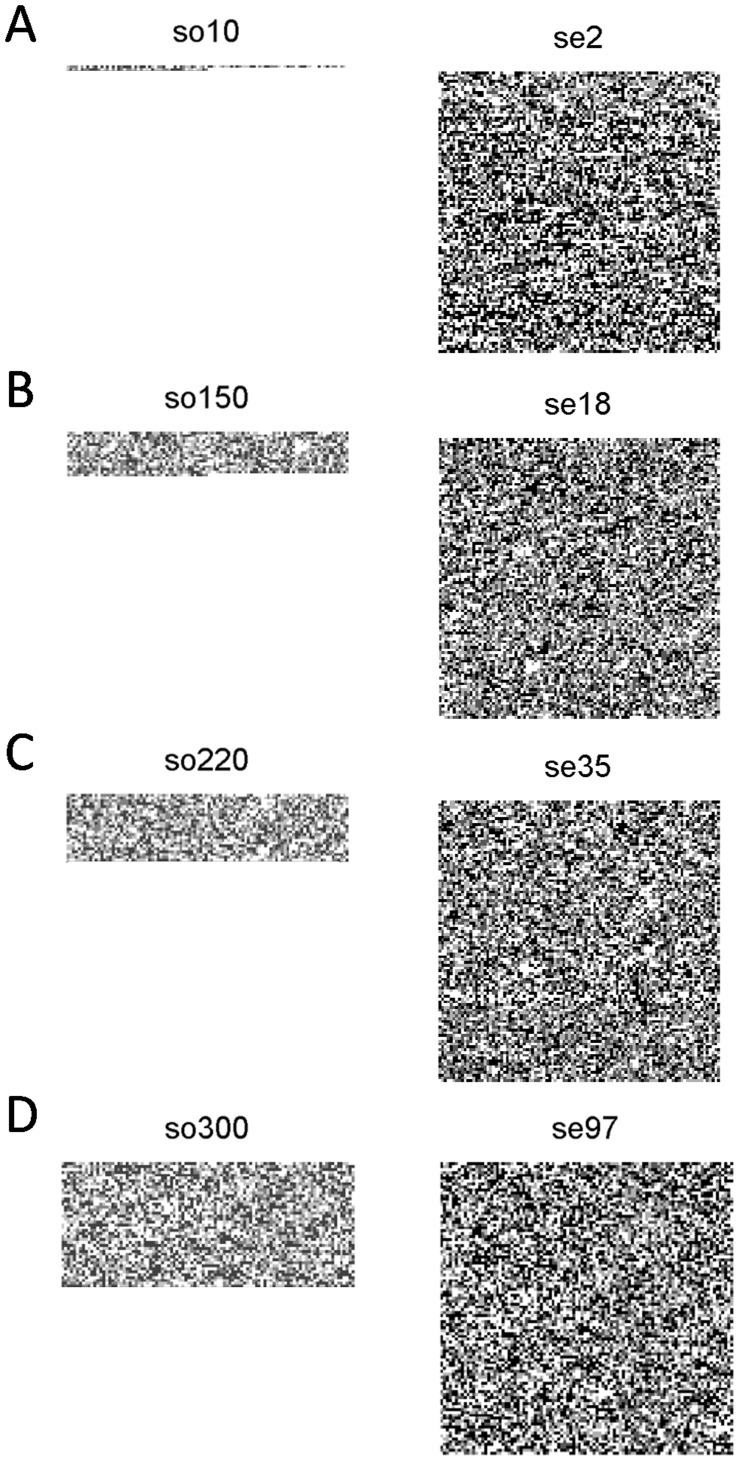
Sequence Codification. The sequences are represented by successions of pixels that compose a *n x m* gray image. The queries or objects (left) have variable lengths while data bases or scenes (right) are represented with a fixed image dimension (*100 x 100* in this example).

Scenes have a fixed dimension (100×100) but query dimension was variable. Figure 5 shows four object sequences, so10, so150, so220 so300 with 150 bp, 1,550 bp, 2,400 bp and 4,300 bp sizes, respectively (left on Figure 5), and four scene sequences, se2, se18, se35, se97 (right of Figure 5). Each object was placed side by side with the scene from where it was extracted. Scenes were the same size while object’s size varied.

#### b) Correlation process

After coding, the next step was to accomplish the object recognition. The Fourier transformation of the object and the scene were carried out and the Vander- Lugt optical correlation process was simulated. A phase only filter was applied to the object, due to its simplicity and good performance [23], but also due to the fact that there is no image distortion in our application, then luminosity is the most relevant aspect. The use of a filter to process the spectral information of the object increases the correlation efficiency [25]. Then, cross-correlation was performed. The cross-correlation theorem (Eq. 2) states that the correlation between two images is the product of their Fourier transformation. Results need to be anti transformed to obtain the spatial peak location.

**Figure 6:**
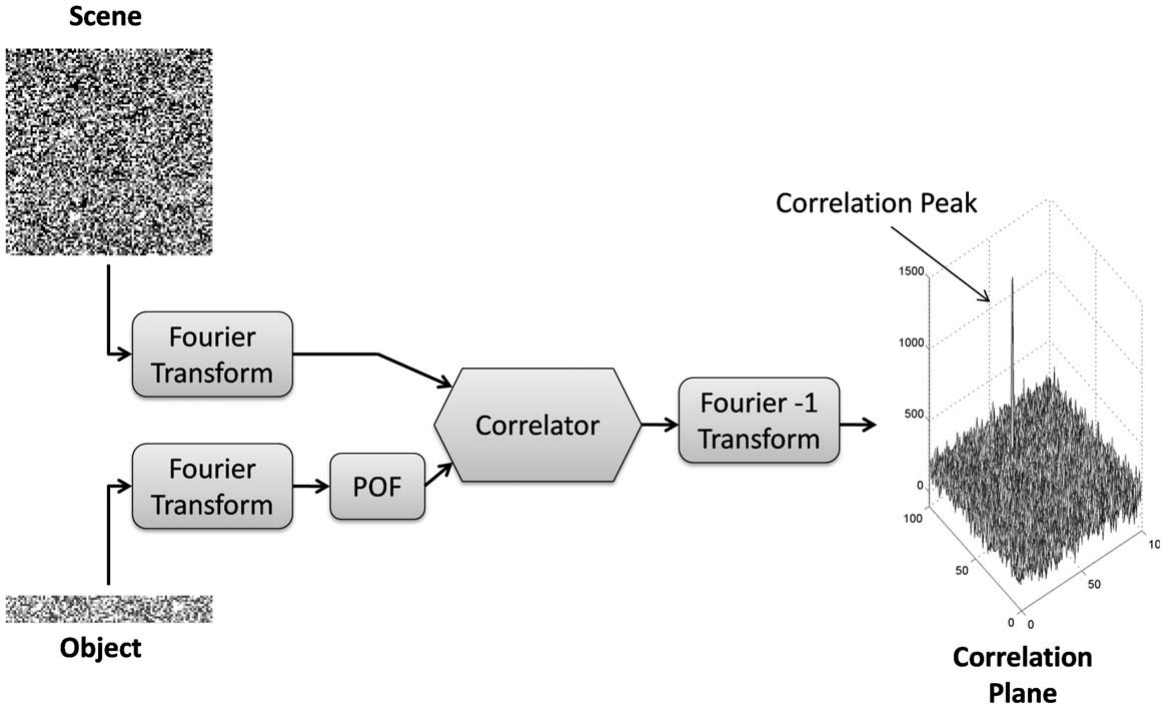
Correlation Process. The scene and object images are Fourier transformed. The object is filtered. Both Fourier Transforms are correlated. The peak indicates matching.

The next step was to identify the maximum peak of intensity at the correlation plane. This was done by identifying the coordinates where the peak intensity of the correlation matrix was maximum. When the peak was identified, a threshold was used to state if the object was or not in the scene. The threshold was established heuristically, after an extensive computed correlation analysis. The threshold was set to 575, which was the value that reached the highest exactitude (Equation 6). Thus, if the peak was above the threshold, the object was stated as present in the scene, and if the peak value was below 575, the object was considered not present in the scene. The whole correlation process is presented in Figure 6.

In this process, as the database was coded in many scenes, each object remained fixed and the scenes changed until the whole database has been correlated and all the peaks are identified. To address eventual span (the object is divided in two scenes), the solution was to overlap the next scene with the previous one. The size of the overlap was the query’s length. This method allows queries search in large databases.

#### c) Evaluation

Correlation process accuracy and efficiency were calculated and compared to BLAST. All objects (original and mutated) were correlated with the scenes. True positives and negatives, false positives and negatives were computed, allowing the four statistical correlation index computations. However, due to the great amount of true negatives, the simulation specificity was too high. This is due to the fact that one object was present in only one of the 100 scenes (one true positive versus 99 true negatives). As exactitude incorporates sensitivity and specificity, the high values of the latter affected it. This is why only sensitivity was used to evaluate the correlation method and compare it with BLAST. The efficiency evaluation was performed measuring the processing time taken by the most important steps of Figure 3 algorithm and compared it with BLAST. The relationship between the time required to perform the correlation and the length of the sequences was also evaluated.

## Results

**Figure 7:**
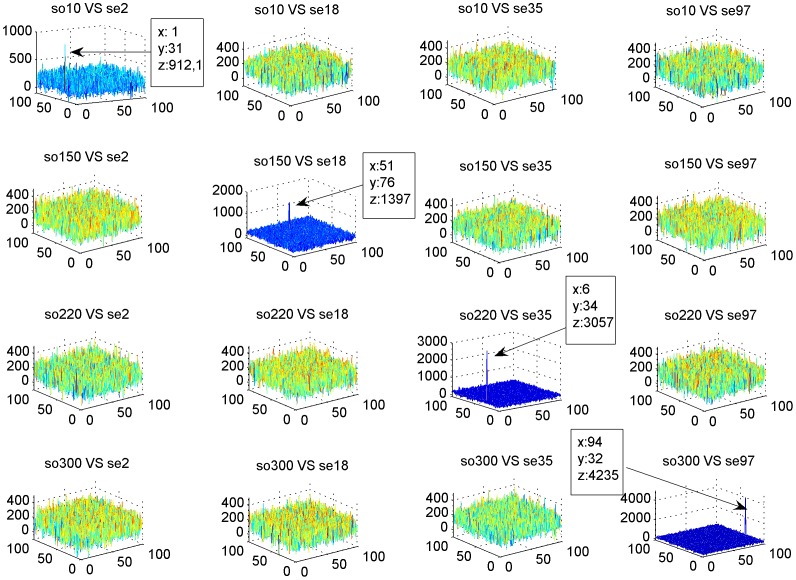
Results of the Correlations. The graphics presents the correlation peaks of the four objects and the four scenes presented in Figure 5. When the object sequence is correlated with the scene from where it was extracted, an outstanding peak can be observed indicating alignment (diagonal). In the other correlations, peaks are less outstanding.

**Figure 8:**
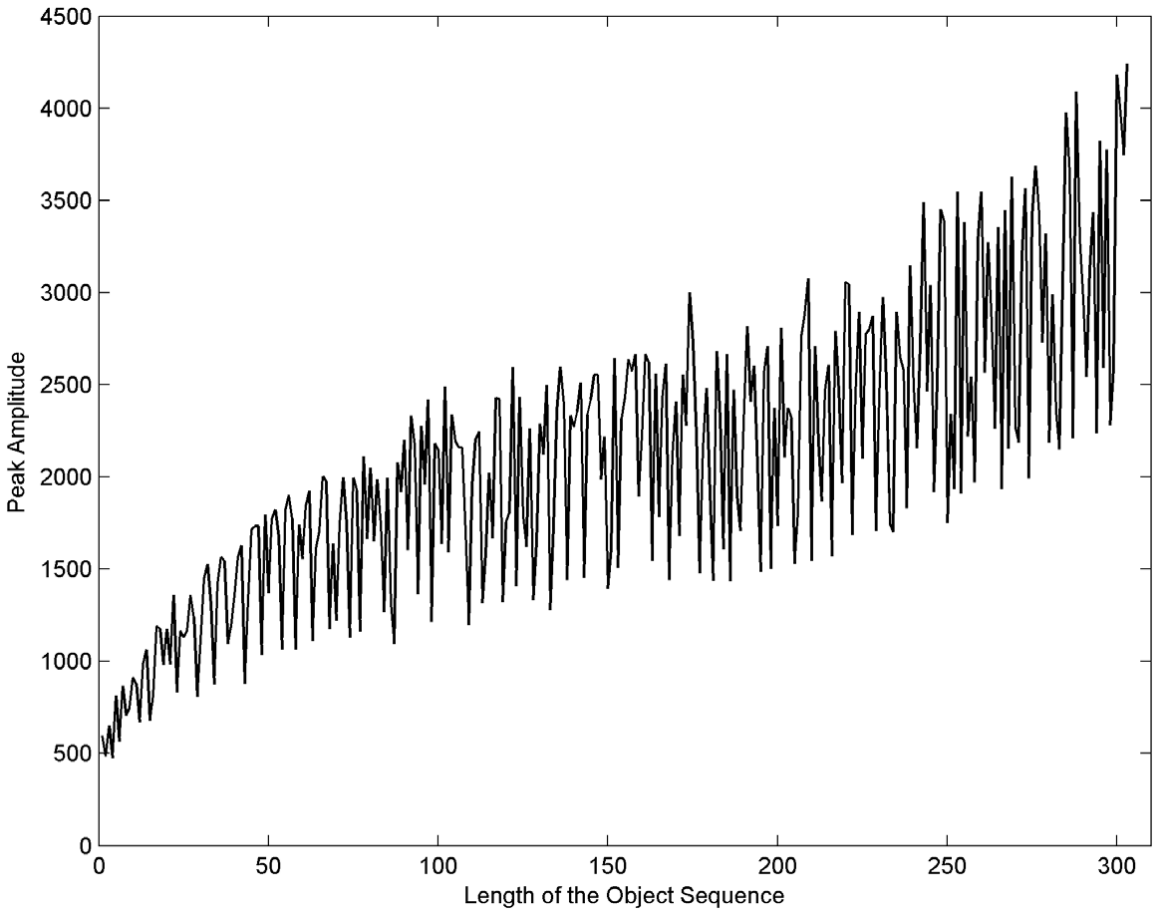
Peak and sequence length relationship . The amplitude of the peak in the correlation plane increases when the sequence length increases; however their relationship is not lineal.

Table 1 shows statistical index results obtained from the simulations. The correlation process showed high levels of sensitivity and specificity even with mutation rates up to 40%. Figure 7 shows the correlation peaks of the four objects and scenes shown in Figure 5. The correlation peak indicates clearly when the object was in its corresponding scene (diagonal in Figure 7). When the object was correlated with other scenes, peaks were less outstanding. In these simulations, the objects had no noise. Another interesting observation is that the peak’s amplitude varied according to the query’s length (Figure 8). The peaks location indicated the objects starting position in the scene. The location was exact most of the time; however, a one pixel shift occurred when the object sequence started in the second half of the scene image. It is important to note that a one pixel shift in the vertical direction corresponds to a 100 base shift. Table 2 shows the percentage of well located object starts and the presence of shift effects for 303 sequences without noise.

**Table 1:** Correlation **s**tatistical indexes (%).

Noise	0%	10%	20%	30%	40%	50%	60%
**Se**	99,01	98,35	97,69	95,05	88,12	42,52	0
**Sp**	98,99	99,98	99,98	99,98	99,98	99,99	99,98
**Ex**	99,98	99,96	99,96	99,93	99,86	99,42	98,98
**Er**	0,02	0,04	0,04	0,04	0,14	0,58	1,02

**Table 2:** Correlation peak relative position e.

	No of objects	Percentage
**Exact location**	191	63,04
**Vertical one pixel shift**	111	36,63
**Wrong location**	1	0,33
**Total**	**303**	**100**

When an object was recognized in a scene, its peak intensity remained a function of the degree of similarity between both sequences. Thus, when the object sequence was longer, the correlation peak was higher, as shown in Figure 8. The same pattern was found for the noise level or “mutation” and the peak’s heights. Higher peaks were associated with lower noise levels and smaller ones with higher noise levels (Table 3).

**Table 3:** Positive peak mean values for several noise levels (%).

Noise	0%	10%	20%	30%	40%	50%	60%
**Intensity**	2111	1806	1503	1211	920	694	0

**Figure 9:**
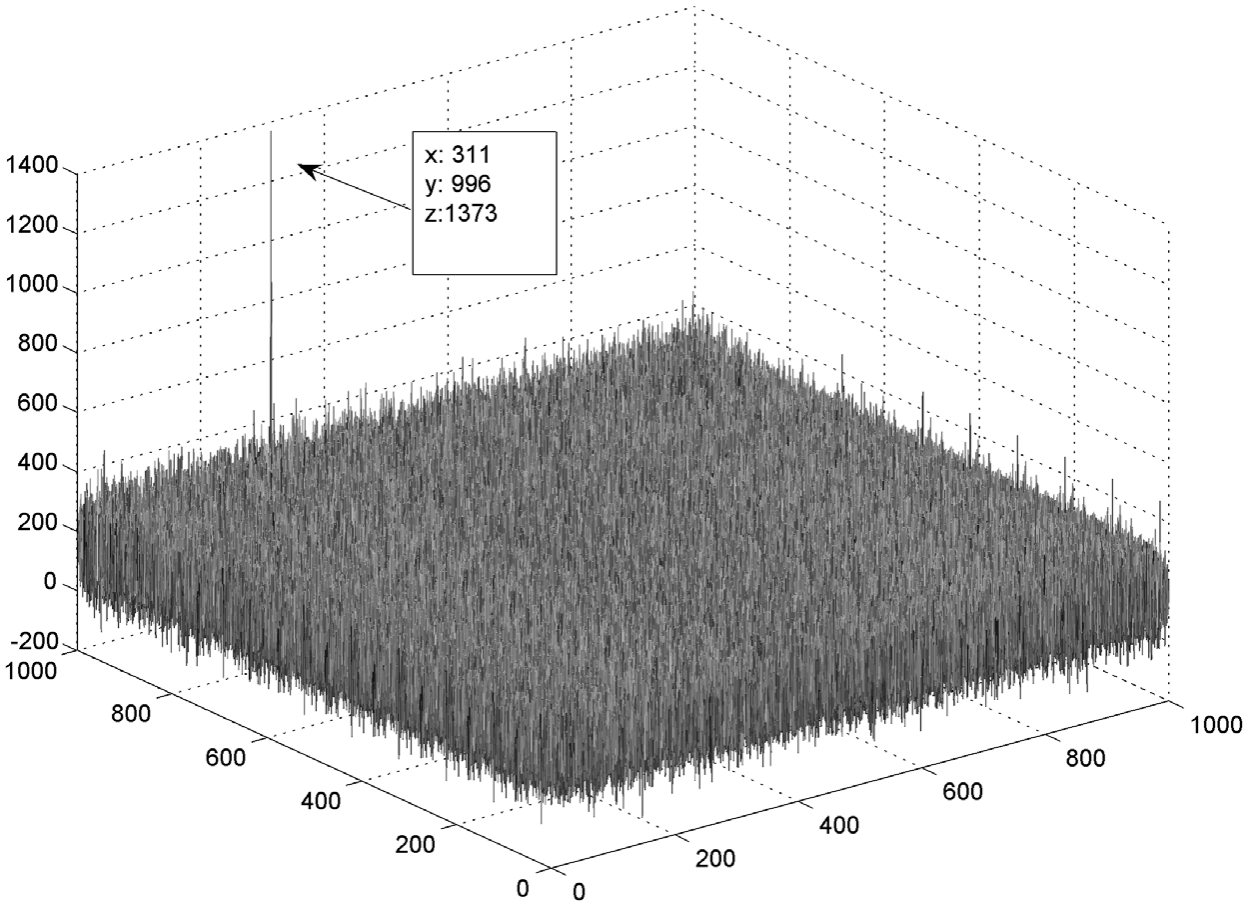
Correlation with great data bases. The figure presents the correlation result of the sequence so33 (400 bases) correlated with a scene that contains the complete database (1000×1000 bases). This shows that the size of the scenes may also vary.

To evaluate the correlation behavior with greater amounts of data, a simulation was carried out considering the correlation of the object so33 (400 bp) with the whole database (1,000,000 bp). Unexpectedly, the peak amplitude of the hit in the 1,000,000 scene was higher than the one obtained for the same object correlated with the 10,000 scene (Figure 9). This is unexpected given that when the whole database is represented in one scene, the pixels are smaller.

Table 4 shows time variation for the main functions used for the correlation in two situations: in the first a 1,000 bp length object was correlated to a 10,000 bp scene; in the second a 4,500 bp length object was correlated to a 10,000,000 bp scene. The results show that transforms and the correlation functions represented more than 70% of the total time. These functions are more sensitive to the sequences length but they can be reduced near to zero when implemented in an optical correlator. The bottle neck of the process is the time taken to load and codify the queries and the scenes. Some optimization alternatives will be analyzed in the Discussion.

**Table 4:** Time (seconds) used by the main functions involved in the correlation process.

Scene’s Length	10.000 pb	10.000.000 pb	Mean	Percentage
Object’s Length	1000 pb	4500 pb		
Textread**	0.672	0.862	0.77	10.2%
Scene Codification	0.532	2.057	1.29	17.3%
Object Codification	0.080	0.133	0.11	1.4%
Correlation	0.747	4.907	2.83	37.7%
FT*	0.234	2.378	1.31	17.4%
Inverse FT*	0.170	2.233	1.20	16.0%
TOTAL	2.435	12.570	7.503	100%

* FT: Fourier Transform.

** Textread: Time taken to import data from disk to a MATLAB variable.

### Comparison with BLAST

**Figure 10:**
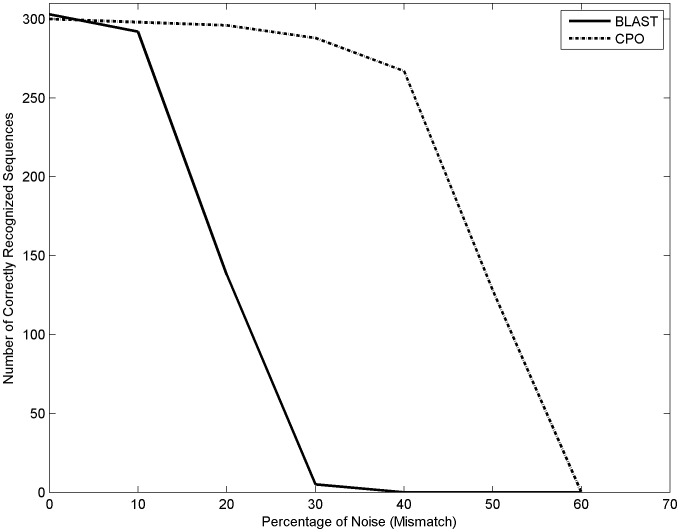
Sensitivity and mutation level. The sensitivity of BLAST decays faster than the sensitivity of the correlation, when the percentage of mutation increases. The latter recognizes similarity with more than 40% of mutated pairs.

BLAST comparisons were focused on two aspects: sensitivity and time. Equation 4 (Se) was applied to BLAST results obtained by blasting the same set of queries and databases previously used with the optical correlator. Table 5 shows sensitivity index values (%) between the correlator and BLAST at the same noise level. Although our optical correlator method and BLAST showed similar sensitivities at low noise levels, BLAST sensitivity rapidly declines when noise increases. In contrast, the correlator remained with high sequence recognition levels, which was translated to high sensitivity levels, even with medium to high levels of noise (40% and 50%; Figure 10). Table 6 shows time, in seconds, taken by both methods to search 303 object sequences in 100 scenes at different noise levels. BLAST was clearly faster than the correlation method and the time involved in the sequence comparisons remained the same at different noise levels.

**Table 5:** Sensitivity (%) of the Correlator and BLAST %.

Noise	0%	10%	20%	30%	40%	50%	60%
**Correlator**	99,01	98,35	97,69	95,05	88,12	42,52	0
**BLAST**	100	96.37	42.90	1.65	0	0	0

**Table 6:** Time (seconds) taken to process 303 objects in 100 scenes.

Noise	0%	10%	20%	30%	40%	50%	60%
**Correlator** *	185.52	259.96	412.97	216.3	269.07	245.13	195.98
**BLAST**	1.01	1.01	1.01	1.01	1.00	1.00	0.99

* Scene Codification time was excluded.

## Discussion

Previous work using sequence alignment correlation methods acknowledged that the main advantage of using Fourier-based approaches compared to standard systems is its higher computational efficiency [20], [25]. In these studies the sequences representation method was linear. The present work’s aim is to evaluate the correlation methods performance and efficiency when each nucleotide is represented as a gray pixel. This representation allows the use of image correlation techniques that can be implemented optically. The image correlation methodology is a robust and well studied process [24]–[32]. Its main weakness occurs when the query object rotates or varies in scale compared to the reference object, decreasing the strength of the correlation output rapidly [24], [25]. In our application there are no such distortions: the compared sequences are coded in the same way, thus the whole potential of the optical image correlation can be exploited.

To evaluate this assumption we compared the correlation methods sensitivity, specificity, and efficiency indexes against a sequence comparison (alignment) method: BLAST. But this comparison is complex: BLAST uses a heuristic local method to evaluate symbol similarity according to statistical significance, whereas the optical correlation method runs an exhaustive 2D-global search. The time used by both methodologies to carry out the alignments was also difficult to compare due mainly to the additional codification step required by the correlation method, and by the fact that the correlation was computer simulated. However, similar to Rockwood et al [7] and Brodzik [23] results, the image correlation method comparison with BLAST was encouraging. When compared to the standard method, the results showed that the correlations performance was similar to BLAST in both situations: when the sequence was present in the database (sensitivity) and when the sequence was absent from the database (specificity). For sequences with mismatch or noise degrees, superior to 20%, the correlation method outperformed BLAST significantly, being able to detect similarities with more than 40% mismatch. Further more, the similarity location is related to the peak’s position, being a good predictor of the object start in the scene. In addition, the correlation peaks amplitude was closely related to the degree of similarity between the two sequences.

The main drawback of the correlation method was the extra time used by the correlation process, especially when compared to BLAST. As shown in Table 4, as the sequence length increases, the correlation step becomes longer. This step can be significantly reduced by carrying out an optical correlation given that transformations and correlations are performed at light speed. However, even in the optical situation, many processes remain digital, like codification, filtering and peak evaluation, representing a bottleneck for the optical process speed. Thus, some alternatives to reduce the time of the digital processes must be studied. For example the codification time can be reduced by having the databases already coded as images. The bases ASCII code can also be used as pixel representation, eliminating the codification step (the ASCCI of A, C, G, T is 65, 67, 71 and 84 respectively; preliminary results showed that changing only C to increase the intervals between base codes gave similar good results). Additionally, the filtering process can be eliminated using the Joint Transform Correlator.

One of the most significant expectations of this work is sequence representation flexibility. In this paper four gray levels were considered to represent each nucleotide base; however, colors may be incorporated. Complementary bases can be represented with closer gray levels to improve sensitivity, sequence substrings can be coded in one pixel (e.g. pairs of bases or codons), different open reading frames [33] can be represented with different colors, etc. On the other hand, as shown in Table 4 and Figure 9, the method can be scaled with greater databases. Both aspects (codification and scene size) allow the processing of great amounts of data as a large number of sequences may be condensed into a substantially smaller representation.

The main contribution of this work was to demonstrate that the image correlation process is able to find similarities between two DNA sequences coded as images, even when the query had a high degree of mutated bases. Further analysis should be performed in particular aspects of the process. For example, it is necessary to associate the amplitude of the correlation peak to the number of matched bases; and thus, creating a score value. It is also necessary to find an equivalent e-value to refine the searches. Rockwood et al [7] proposed a base-by-base comparison after the peak is detected to extract the exact matches. Thus a combination of global and local search algorithms can be studied. The shift of the correlation peak location that occurs when the sequence is in the second half of the scene seems to be constant, thus new simulations should be performed to evaluate if a constant criterion can be used to adjust the location according to the horizontal position of the peak. These issues have to be explored before applying the correlation method to real data.

The main purpose of this work was to evaluate the performance of image correlation processes applied to DNA sequence alignment and to compare it to one of the most popular alignment alternatives, BLAST. The optical correlator was computer simulated and 2121 sequences with different mismatch lengths and levels were searched in 100 sequences of 10000 bases each. The results showed that image correlation had a high sensitivity, specificity and exactitude when recognizing similarities between sequences. This ability was superior to BLAST when the percentage of mismatches increased. The high point of the correlation peak was related to the degree of similarity between the sequences. Its location indicated the spatial location of the match. The time efficiency of the method was very low compared to BLAST, due to the fact that codification and correlations were performed in a computer. However the results are encouraging as some processing could be done optically, thus reducing hugely the processing time.

Future work includes to decrease the time of the algorithms that will remain digital, to create score and e-value equivalent indexes and to test the process in a real optical correlator. In our opinion, the method opens possibilities for future developments, mainly focused on the pixel representation of one or more bases, to increase the amount of data analyzed during one correlation and decrease search time. The results obtained in this work support the idea that optical image correlation may be an interesting research alternative to face the challenge of processing the large amount of genomic data currently accumulating from most genomic researches.
